# Supplementary figures and images for: The changing impact of the active job openings-to-applicants ratio (AJOAR) on ambulance dispatches during deflation: A longitudinal ecological study
Source: PLoS One. 2025 May 28;20(5):e0320914. doi: 10.1371/journal.pone.0320914 (PMC12118969; doi:10.1371/journal.pone.0320914)

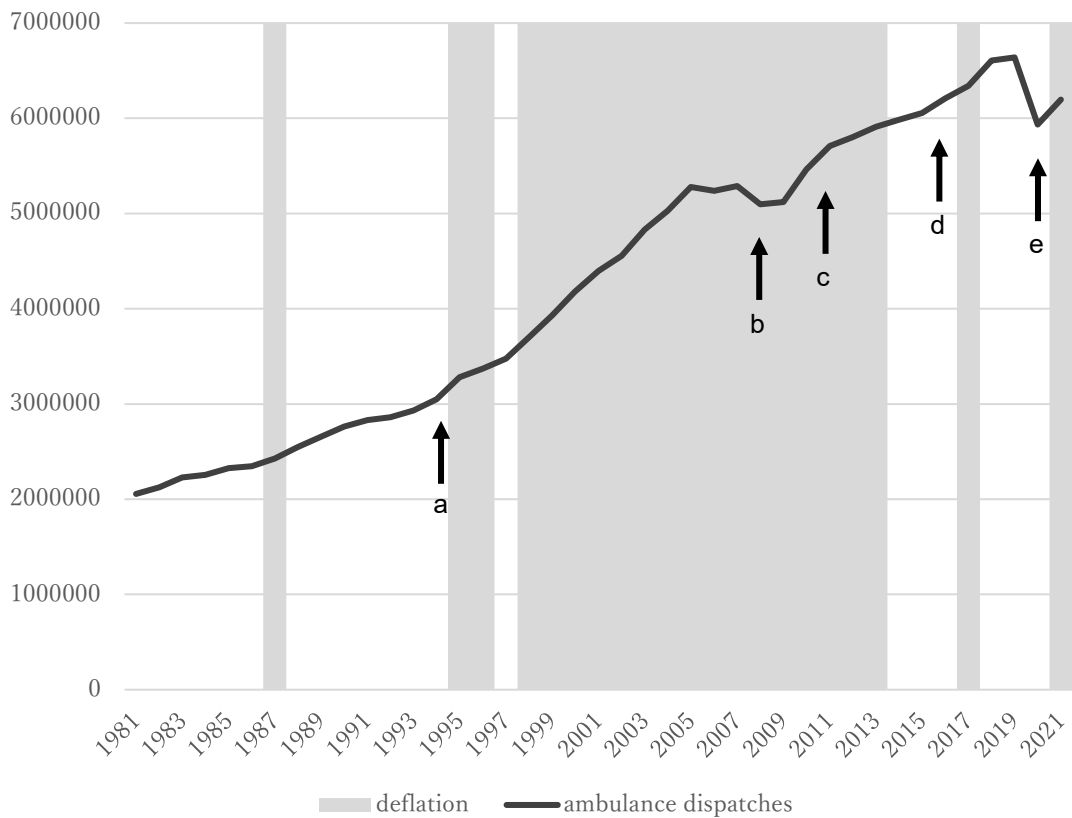

Supplement: S1 Fig — Arrows indicate major socioeconomic events suffered by Japan of (a) the economic bubble burst, (b) the global financial crisis, (c) the Great East Japan Earthquake, (d) the introduction of the additional fee for a first-time patient without a referral, and (e) the COVID-19 pandemic. (PDF) [file pone.0320914.s001.pdf]

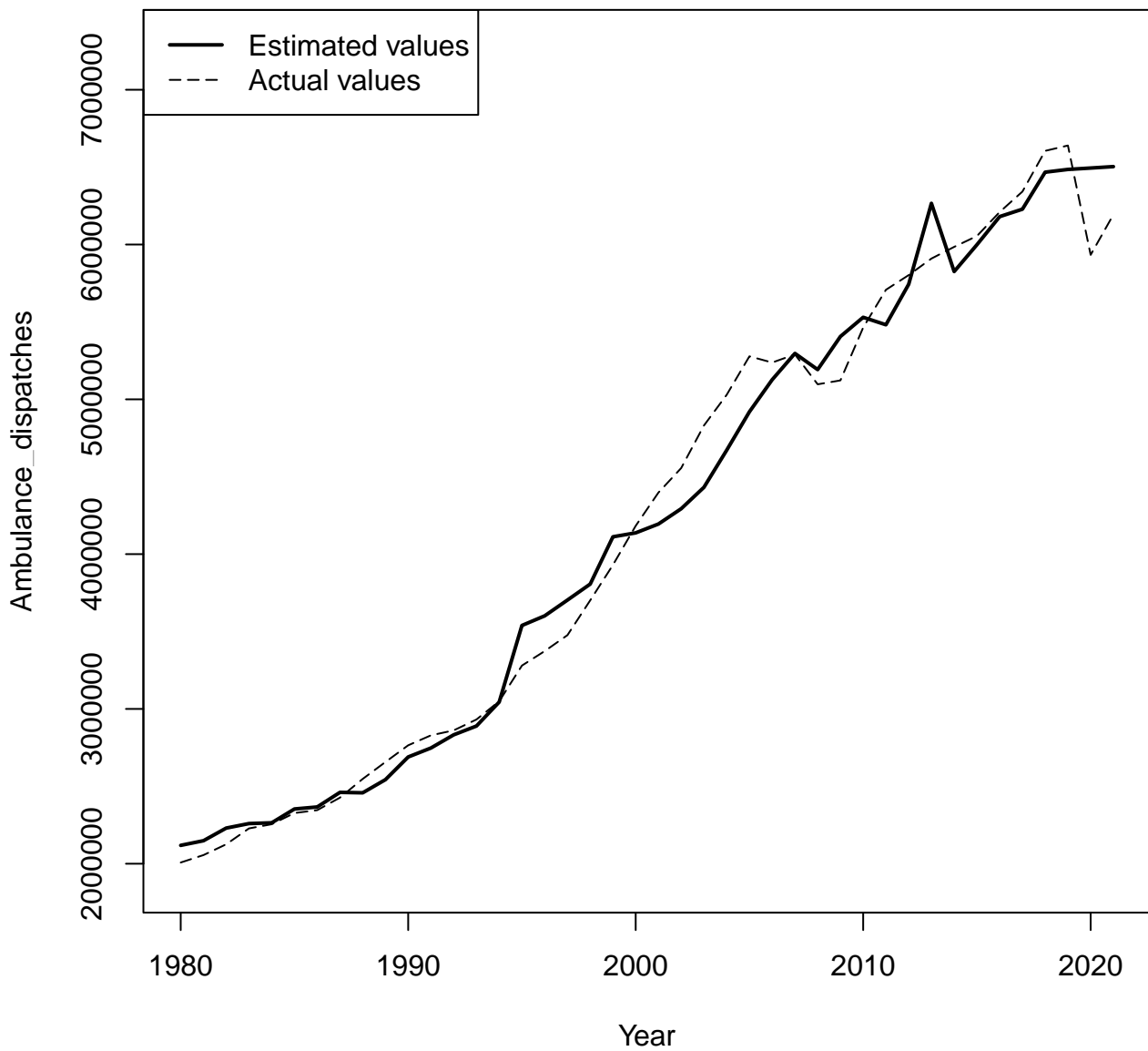

Supplement: S2 Fig — The regression model consists of the older population, mean temperature, Tokyo Stock Price Index during deflation, and Tokyo Stock Price Index during inflation, including the total population as an offset variable. (PDF) [file pone.0320914.s003.pdf]

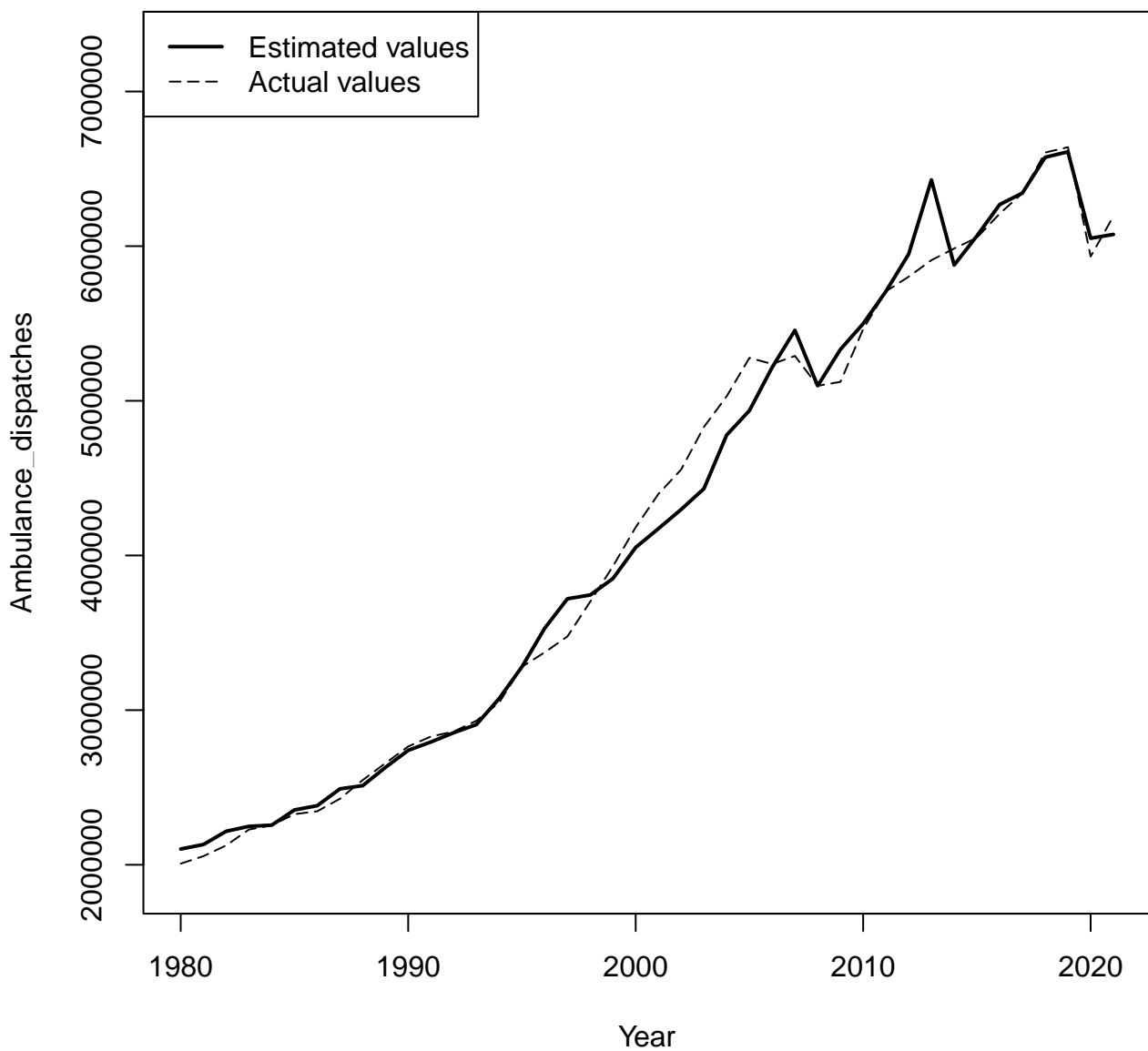

Supplement: S3 Fig — The regression model consists of the older population, mean temperature, active job openings-to-applicants ratio during deflation, active job openings-to-applicants ratio during inflation, the economic bubble burst, the global financial crisis, the Great East Japan Earthquake, the introduction of the additional fee for a first-time patient without a referral, and the COVID-19 pandemic, including the total population as an offset variable. (PDF) [file pone.0320914.s005.pdf]
