# Supplementary material for: The changing impact of the active job openings-to-applicants ratio (AJOAR) on ambulance dispatches during deflation: A longitudinal ecological study
Source: PLoS One. 2025 May 28;20(5):e0320914. doi: 10.1371/journal.pone.0320914 (PMC12118969; doi:10.1371/journal.pone.0320914)
Supplement: S1 Table — GEE: generalized estimation equation; CI: confidence interval; AJOAR: active job openings-to-applicants ratio; TOPIX: Tokyo Stock Price Index; QIC: quasi-likelihood under the independence model criterion. * P < .05. The regression model of main analysis consists of the older population, mean temperature, AJOAR during deflation, and AJOAR during inflation, including the total population as an offset variable. The regression model of sensitivity analysis consists of the older population, mean temperature, TOPIX during deflation, and TOPIX during inflation, including the total population as an offset variable. (DOCX) [file pone.0320914.s002.docx]

**S1 Table. Comparison of results from the main and sensitivity analyses for annual ambulance dispatches.**

|  | Main analysis  GEE estimate (95% CI) | Sensitivity analysis  GEE estimate (95% CI) |
| --- | --- | --- |
| AJOAR during deflation | 0.165 (0.087 to 0.243) * |  |
| AJOAR during inflation | 0.019 (−0.021 to 0.059) |  |
| TOPIX during deflation, 10^3^ |  | 0.067 (0.021 to 0.113) * |
| TOPIX during inflation, 10^3^ |  | −0.015 (−0.046 to 0.016) |
| Older population, 10^7^ people | 0.400 (0.381 to 0.418) * | 0.413 (0.396 to 0.429) * |
| Low-temperature effect | 0.021 (−0.006 to 0.048) | 0.017 (−0.017 to 0.050) |
| QIC | −5,070,716,387 | −5,070,693,572 |

GEE: generalized estimation equation; CI: confidence interval; AJOAR: active job openings-to-applicants ratio; TOPIX: Tokyo Stock Price Index; QIC: quasi-likelihood under the independence model criterion. * P <.05. The regression model of main analysis consists of the older population, mean temperature, AJOAR during deflation, and AJOAR during inflation, including the total population as an offset variable. The regression model of sensitivity analysis consists of the older population, mean temperature, TOPIX during deflation, and TOPIX during inflation, including the total population as an offset variable.
