# Supplementary material for: The changing impact of the active job openings-to-applicants ratio (AJOAR) on ambulance dispatches during deflation: A longitudinal ecological study
Source: PLoS One. 2025 May 28;20(5):e0320914. doi: 10.1371/journal.pone.0320914 (PMC12118969; doi:10.1371/journal.pone.0320914)
Supplement: S2 Table — GEE: generalized estimation equation; CI: confidence interval; AJOAR: active job openings-to-applicants ratio; QIC: quasi-likelihood under the independence model criterion. * P < .05. The regression model of main analysis consists of the older population, mean temperature, AJOAR during deflation, and AJOAR during inflation, including the total population as an offset variable. The regression model of extended analysis builds on this by additionally including major socioeconomic events: the economic bubble burst, the global financial crisis, the Great East Japan Earthquake, the introduction of the additional fee for a first-time patient without a referral, and the COVID-19 pandemic, while still using the total population as an offset variable. (DOCX) [file pone.0320914.s004.docx]

**S2 Table. Results of the main analysis and the extended analysis including major socioeconomic events for the annual ambulance dispatches.**

|  | Main analysis  GEE estimate (95% CI) | Extended analysis  GEE estimate (95% CI) |
| --- | --- | --- |
| AJOAR during deflation | 0.165 (0.087 to 0.243) * | 0.142 (0.066 to 0.217) * |
| AJOAR during inflation | 0.019 (−0.021 to 0.059) | 0.007 (−0.037 to 0.051) |
| Older population, 10^7^ people | 0.400 (0.381 to 0.418) * | 0.412 (0.394 to 0.430) * |
| Low-temperature effect | 0.021 (−0.006 to 0.048) | 0.019 (−0.007 to 0.045) |
| Economic bubble burst |  | −0.040 (−0.063 to −0.017) * |
| Global financial crisis |  | −0.066 (−0.100 to −0.031) * |
| Great East Japan Earthquake |  | 0.017 (−0.010 to 0.044) |
| Additional fee for a first-time patient without a referral |  | 0.002 (−0.030 to 0.034) |
| COVID-19 pandemic |  | −0.088 (−0.120 to −0.056) * |
| QIC | −5,070,716,387 | −5,070,814,552 |

GEE: generalized estimation equation; CI: confidence interval; AJOAR: active job openings-to-applicants ratio; QIC: quasi-likelihood under the independence model criterion. * P <.05. The regression model of main analysis consists of the older population, mean temperature, AJOAR during deflation, and AJOAR during inflation, including the total population as an offset variable. The regression model of extended analysis builds on this by additionally including major socioeconomic events: the economic bubble burst, the global financial crisis, the Great East Japan Earthquake, the introduction of the additional fee for a first-time patient without a referral, and the COVID-19 pandemic, while still using the total population as an offset variable.
