# Supplementary material for: The changing impact of the active job openings-to-applicants ratio (AJOAR) on ambulance dispatches during deflation: A longitudinal ecological study
Source: PLoS One. 2025 May 28;20(5):e0320914. doi: 10.1371/journal.pone.0320914 (PMC12118969; doi:10.1371/journal.pone.0320914)
Supplement: Attachment — Submitted filename: Response_Letter.docx [file pone.0320914.s007.docx]

Dr. Emily Chenette,

Editor-in-Chief

PLOS ONE

Dear Dr. Chenette,

We would like to thank you and the reviewers for your consideration and comments on our manuscript. We have revised the manuscript accordingly and have provided a point-by-point response to the comments below.

We hope that our manuscript will now be suitable for publication in your journal.

Kindest regards,

Yohei Kamikawa

Department of Emergency Medicine, University of Fukui Hospital, Fukui, Japan

E-mail: fundarike.ykami@gmail.com

Dear Editor,

Thank you very much for your careful consideration of our article.

**Question:** *Please ensure that your manuscript meets PLOS ONE's style requirements, including those for file naming. The PLOS ONE style templates can be found at*

*https://journals.plos.org/plosone/s/file?id=wjVg/PLOSOne_formatting_sample_main_body.pdf and*

*https://journals.plos.org/plosone/s/file?id=ba62/PLOSOne_formatting_sample_title_authors_affiliations.pdf*

**Answer:** We agree that our manuscript should comply with the requirements. We have carefully reviewed the templates and revised the format accordingly (Lines 12, 14, 31, 32, 62, 68, 69, 81, 135, 156, 201, 202, 215, 291, 292, 323, 355, 379-380, 399, 413, 435, 442, 446, 536).

Dear Reviewer #1,

Thank you very much for your careful consideration of our article.

**Question:** *1. Explanatory Variables*

*The use of AJOAR (Active Job Openings-to-Applicants Ratio) as an explanatory variable for ambulance dispatches is somewhat unconventional. While AJOAR is typically employed to explain labor market trends, its causal link to ambulance dispatches remains unclear. The mechanism through which labor market conditions—specifically the balance between job offers and job seekers—impact emergency medical service demand needs further elaboration. Even if there is a hypothesis suggesting that economic instability and stress may indirectly influence health and lead to increased ambulance usage, this connection should be better justified, perhaps by incorporating other macroeconomic factors such as the unemployment rate or GDP growth.*

**Answer:** We agree that additional macroeconomic indicators should be examined to confirm that macroeconomic trends influence the number of ambulance dispatches. Therefore, we conducted sensitivity analyses by replacing AJOAR with TOPIX, a stock price index, in the main analyses. The results were consistent with those of the main analyses. Consequently, as different macroeconomic indicators from varying perspectives yielded similar results, it can be concluded that macroeconomic trends—rather than the conditions of the labor or stock markets—are associated with the number of ambulance dispatches. These arguments have been added to the manuscript (Lines 92-93, 167-169, 209-210, 212-213, 224-230, 251-252, 293, 295-301, S1 Table, S2 Fig). The discussion on how macroeconomic trends influence the number of dispatches is already included in the subsection "Deflation impact" within the Discussion section, with references to previous studies (Lines 323-353).

**Question:** *2. Insufficient Consideration of Socioeconomic Events*

*Significant socioeconomic events such as the bursting of Japan’s bubble economy, the Lehman shock, the Great East Japan Earthquake, and the COVID-19 pandemic likely had considerable impacts on ambulance dispatches. By not accounting for these events, there is a risk of bias in the results. Including these events as adjustment factors could lead to a more robust model. Could you consider incorporating these into your analysis?*

**Answer:** We acknowledge that incorporating socioeconomic events could potentially lead to more robust conclusions. However, when the number of explanatory variables exceeds four—one-tenth of the total number of outcome points (42 years)—the validity of the GEE analysis may be compromised. Therefore, we performed the analyses as an extended analysis for reference. As a result, only events with nationwide economic impact were found to be statistically significant alongside AJOAR during deflation. These points have been added to the manuscript (Lines 185-189, 280-289, 366-377, S2 Table, S3 Fig).

**Question:** *3. Regional Differences*

*While this study utilizes data from across Japan, it is expected that ambulance dispatch patterns and economic conditions differ between urban and rural areas. The exclusion of factors such as regional population density, medical access, and differing attitudes towards emergency medical services may limit the generalizability and accuracy of your model. A subgroup analysis that incorporates regional data could strengthen the robustness of the findings. Is it feasible to attempt such an analysis?*

**Answer:** We acknowledge that subgroup analyses incorporating regional data could have provided more robust conclusions. However, as mentioned in the Limitations section (Lines 422-424), such analyses were not feasible due to the unavailability of gross prefectural deflator data prior to 1995. Nevertheless, this may limit the generalizability of the study. Therefore, we added a statement in the Limitations section to acknowledge the possibility of unknown factors beyond the variables examined in our model, which may have influenced the results (Lines 419-420).

**Question:** *4. Simplistic Treatment of Temperature*

*The paper uses the annual average temperature as a single explanatory variable, but ambulance dispatches may be heavily influenced by seasonal variations (e.g., the winter flu season). Could you elaborate on the appropriateness of your current method for adjusting for temperature as a confounding factor?*

**Answer:** We acknowledge that the annual mean temperature may not capture seasonal variations. However, this study follows the methodology of a previous study that demonstrated a significant impact of mean temperature on the number of ambulance dispatches. Excluding this variable from our model would have been overly arbitrary, so we decided to retain it. These considerations have been added to the Methods section (Lines 111-116).

**Question:** *5. Model Explanatory Power*

*The study employs 42 years of data in its regression analysis, but further discussion is needed regarding the model’s explanatory power. For example, the paper mentions that the model fit deteriorates after 2013, but there is no clear explanation for this. Additionally, while a sensitivity analysis was conducted, the discussion of whether the chosen model is truly optimal remains somewhat vague. Could you clarify these points?*

**Answer:** We agree that the explanatory power of the models should be discussed further. To address this, we added Goodness-of-Fit tests to the statistical analyses. These tests confirmed that there were no significant issues with the model’s fit. This information has been incorporated into the manuscript (Lines 165-167, 222-223, 228-229, 232-233, 286-287).

Regarding the apparent poor fit around 2013, we consider that this is due to the oversimplified dichotomous classification of periods as either deflationary or inflationary, as described in the manuscript (Lines 385-386). Specifically, during the transition from deflation to inflation, when the AJOAR consistently increases, the regression estimates in our model show a sharp decline. This occurs because AJOAR peaks just before the transition during deflation and then abruptly drops to zero at the onset of inflation. This explains the deviation observed around 2013. We have added this explanation to the Discussion section (Lines 386-390).

As for whether our model is truly optimal, we acknowledge the possibility of unknown variables and more refined models. Since our model was based on a previous study, it may not represent the most optimal approach. We have added this consideration to the manuscript (Lines 114-116, 419-420).

Dear Reviewer #2,

Thank you very much for your careful consideration of our article.

**Question:** *1. There are many influential factor to individual outcome (ambulance dispatch in this study), however, an ecological study can not handle these individual-level factors such as age, sex, comorbidity, ADL, and other lifestyles. Thus, employing multivariable analysis with the aggregated data cannot provide any meanings.*

**Answer:** We acknowledge that analyzing individual-level factors would be ideal. However, such analyses were not possible due to the fully anonymized nature of the data provided by government offices. As a result, we used aggregated data to capture general population trends in Japan, which allowed us to obtain meaningful and beneficial findings. This discussion has been added to the Limitations section (Lines 426-427, 428-430).

With regard to multivariable analyses using aggregated data, we have previously conducted similar analyses, and the results were published in a peer-reviewed journal:

Reference: Kamikawa Y. Impact of the active job openings-to-applicants ratio on the number of ambulance dispatches in Japan, 2003–2021: a longitudinal ecological study. BMJ Open. 2024;14:e083755.

Additionally, since numerous studies have employed multivariable analyses with aggregated data, we believe there are no methodological issues with our approach. Examples include:

Reference: Yazaki H, Nishiura H. Ambulance Transport of Patients with Mild Conditions in Hokkaido, Japan. Int J Environ Res Public Health. 2020 Feb 2;17(3):919.

Reference: Ikeda N, Fuse K, Nishi N. Changes in the effects of living with no siblings or living with grandparents on overweight and obesity in children: Results from a national cohort study in Japan. PLoS One. 2017 Apr 17;12(4):e0175726.

**Question:** *2. about Table 2.*

*Line 191 stated that AJOAR varies from 0.63 to 1.09. Regression coefficients of AJOAR (table 2) looks extremely small so that there is almost no relationship with the outcome.*

*In addition, the coefficient shows opposite (negative) in sensitivity analysis. This means the coefficient is unstable in this study.*

**Answer:** While the GEE coefficient estimate of 0.165 may appear small, its real-world impact is substantial. We have added this clarification to the Discussion section (Lines 303-312).

Regarding the term “sensitivity analysis,” we acknowledge that this terminology was inappropriate. The purpose of these analyses was not to evaluate the robustness of the model but rather to compare the fit between our current model and a previous study’s model. Therefore, the term “secondary analysis” would have been more accurate. We have revised this terminology throughout the manuscript.

Considering this clarification, the coefficient estimate of AJOAR during deflation was not unstable. The secondary analyses were not designed to test minor variations in conditions but rather to assess a fundamental change in the hypothesis—specifically, whether the contribution of AJOAR varies between inflationary and deflationary periods. For example, if we were investigating whether a plant releases more oxygen or carbon dioxide, the result might indicate less oxygen depending on the observation period. However, if we were testing the hypothesis that a plant’s gas emissions differ between daytime and nighttime, we could find that it releases more oxygen during the day. Should we conclude that the amount of oxygen released is unstable because of a negative result in the secondary analysis? The answer is “no.” In this case, we should conclude that the hypothesis of constant oxygen and carbon dioxide emissions is incorrect, and instead, a plant produces more oxygen during the day and less at night. We hope this analogy helps clarify that our study employed a similar type of analysis, focusing on testing changes in the underlying hypothesis.

The significant negative estimate for the GEE coefficient in the secondary analysis was likely a misleading result caused by a fundamental flaw in the hypothesis that the contribution of AJOAR remained constant regardless of whether the period was inflationary or deflationary. The p-value is known to be unreliable when the hypothesis is incorrect.

Reference: Price R, Bethune R, Massey L. Problem with p values: why p values do not tell you if your treatment is likely to work. Postgrad Med J. 2020 Jan;96(1131):1-3. doi: 10.1136/postgradmedj-2019-137079. Epub 2019 Oct 29. PMID: 31662411.

Since relying solely on p-values can lead to misinterpretation, additional evidence is necessary to avoid such errors. In this study, we included graphical confirmation to validate the findings. The graph from the main analysis aligned well with the actual trend, whereas the graph from the secondary analysis failed to reflect the observed trend. Based on this, we could conclude that AJOAR contributed to the number of ambulance dispatches only during deflationary periods. These points have been added to the Discussion section (Lines 313-317).

**Question:** *3. The author should not refer to non-accepted paper(Research Square).*

**Answer:** Fortunately, the paper was recently accepted and published in BMJ Open. We have added it to the References section (Lines 472-474).

Dear Reviewer #3,

Thank you very much for your careful consideration of our article.

**Question:** *1. In this period, I know that nationwide pre-hospital telephone consultations and assistance through online medical systems are coming in later in this time setting, but is that impact related to this ambulance mobilization because of pre-hospital consultation services are being established as a policy of the local government?*

**Answer:** We recognize that pre-hospital telephone consultations and online medical assistance systems may influence the number of ambulance dispatches. However, some municipalities have yet to implement these services. Even among those that have, the timing of implementation varies. Therefore, accounting for such factors is not appropriate in this study, which focuses on nationwide trends affecting dispatch numbers. We acknowledge that conducting subgroup analyses incorporating regional data could have strengthened our conclusions. However, as noted in the Limitations section (Lines 422-424), such analyses were not feasible due to the unavailability of gross prefectural deflator data before 1995. Nevertheless, this limitation may affect the generalizability of our findings. To address this, we added a statement in the Limitations section acknowledging the potential influence of unknown factors not examined in this study (Lines 419-420).

**Question:** *2. In your conclusions, 'This study demonstrated that the AJOAR during deflation was associated with increased ambulance dispatches.' Are there any specific measures that the government is trying to put in place to address this issue? If you know, please le us know.*

**Answer:** Some argue that Japan's deflation, which began in 1995, was driven by inappropriate monetary and fiscal policies that contradicted economic principles, and that proper policies aligned with contemporary economic theories could have prevented it. However, we believe that exploring which policies might mitigate deflation falls outside the scope of this study. This research focuses exclusively on the relationship between ambulance dispatch numbers and macroeconomic indicators. Therefore, we have refrained from commenting on matters not directly supported by our findings. This perspective is addressed in the Discussion section (Lines 409–411). If your question pertains to what the government should prioritize during deflation, the focus must be on strengthening and optimizing emergency medical services. That said, the exploration of specific responses to such scenarios also lies beyond the scope of this study. Accordingly, we have deliberately avoided discussing issues not substantiated by our results. This point has been also highlighted in the Discussion section (Lines 406–411).

**Question:** *3. Regarding the current analysis, the association between economic status and emergency ambulance transportation in an analysis conducted in Japan, a country with a health insurance system in which emergency ambulance transportation is free of charge; would this trend change if the ambulance service were in a country where it is paid for? Also, what do you think will happen if ambulances are paid for in Japan in the future, or if certain standards for ambulance transport are set?*

*Do you think that the trend of ambulance transports will continue to increase during deflation?*

*Because if the world economy is moving in the direction of deterioration and is not easily turned toward inflation, do you think there are any measures that can be taken to overcome this situation?*

**Answer:** We believe it is inappropriate to draw conclusions about other countries or time periods with ambulance systems different from that of present-day Japan, as this study is based solely on data from the current Japanese context. This limitation has been clarified in the Limitations section (Lines 414-416).

Based on the findings of this study, whether the number of ambulance dispatches is likely to increase if deflation persists depends on trends in the AJOAR and the older population.

If deflation is unavoidable, the government must prioritize strengthening and optimizing emergency medical services. However, exploring potential responses to a deteriorating global economy lies beyond the scope of this research. Therefore, we have deliberately avoided addressing issues not directly supported by our findings. This point has been included in the Discussion section (Lines 406–411).

Dear Reviewer #4,

Thank you very much for your careful consideration of our article.

**Question:** *1. Although authors excluded both individual and rejonal factors from variables, I think the numbers of variables is relatively small. I feel that the number of doctors (especially emergency physicians), emergency institutions, family structure and elderly care facility residents might be related to the number of long term ambulance dispatchs.*

**Answer:** We recognize that incorporating regional and individual data into analyses may have strengthened our conclusions. However, as noted in the Limitations section (Lines 422-424), such analyses were not feasible due to the lack of gross prefectural deflator data before 1995. Additionally, when the number of explanatory variables exceeds four—one-tenth of the total outcome points (42 years)—the validity of the GEE analysis can be compromised (Lines 366-369). Consequently, we structured our model as described in the manuscript. Nonetheless, this approach may limit the study's generalizability. To address this, we added a statement to the Limitations section acknowledging the potential impact of factors outside the scope of the variables examined in this study (Lines 419–420).

**Question:** *2. The authors considered that mean temperature was not a significant relevant variable, which may be due to the use of annual data. I think it is not appropriate to use mean temperature as a variable.*

**Answer:** We recognize that using the annual mean temperature as a variable is not ideal, as it fails to account for seasonal variations. However, this study builds on the methodology of a prior study that identified a significant relationship between mean temperature and the number of ambulance dispatches. Removing this variable from our model would have been overly arbitrary, so we chose to include it. These points have been addressed in the Methods section (Lines 111–116).
