# Supplementary material for: The changing impact of the active job openings-to-applicants ratio (AJOAR) on ambulance dispatches during deflation: A longitudinal ecological study
Source: PLoS One. 2025 May 28;20(5):e0320914. doi: 10.1371/journal.pone.0320914 (PMC12118969; doi:10.1371/journal.pone.0320914)
Supplement: Attachment — Submitted filename: Response_Letter_auresp_2.docx [file pone.0320914.s008.docx]

Dr. Emily Chenette,

Editor-in-Chief

PLOS ONE

Dear Dr. Chenette,

We would like to thank you and the reviewers for your consideration and comments on our manuscript. We have revised the manuscript accordingly and have provided a point-by-point response to the comments below.

We hope that our manuscript will now be suitable for publication in your journal.

Kindest regards,

Yohei Kamikawa

Department of Emergency Medicine, University of Fukui Hospital, Fukui, Japan

E-mail: fundarike.ykami@gmail.com

Dear Editor,

Thank you very much for your careful consideration of our article.

**Question:** *1. While I acknowledge that ecological study is an established study design that provides a certain level of meaningful evidence, as the authors have responded, I also recognize that this design has substantial limitations, such as ecological fallacy, which aligns with Reviewer 2's concerns. Therefore, I strongly believe the authors need to add appropriate discussion about the limitations inherent to the study design.*

**Answer:** We agree that the limitations related to the study design should be discussed in more detail. In the Limitations section, we have added a caution regarding the ecological fallacy, emphasizing that the results should not be interpreted as evidence of individual-level causation but remain valid in the context of population-level trends. (Lines 422-431)

**Question:** *2. I agree with Reviewer 4's point to the initial submission regarding the relatively small number of variables. There may be residual confounding, and the results could be biased. Therefore, the authors should discuss how this limited number of variables might affect the results and what aspects readers should consider when interpreting them. In the current form, the authors state: "Second, the number of explanatory variables was limited in order to maintain the reliability of the regression analyses... Nevertheless, this study effectively captured the trend in ambulance dispatches despite its concise model structure." While this description acknowledges the limitation, the authors claim the results are not problematic without providing adequate rationale or discussion.*

**Answer:** We agree that the limitations due to the small number of variables should be discussed. First, we removed any wording that could be misinterpreted as implying that the results were free of issues. While we have already explained the rationale for variable selection in the “Other factors” section (Lines 135-159), we have now clarified the possibility of residual confounding and variable selection bias in the Limitations section. (Lines 435-438)

**Question:** *3. Regarding the fundamental methodology, including variable selection, the validity of "mean" temperature, and the validity of the simple model (with few variables), the authors have cited their own previous study (reference 11). While I acknowledge that reference 11 is clinically important, for methodological issues, the authors should provide additional references from other researchers demonstrating that these methods are widely accepted and valid, such as methodological studies or similar research papers.*

**Answer:** We agree that a more detailed methodological discussion is necessary.

Regarding variable selection, we have added a methodological article on ecological studies as reference 26. This article emphasizes the importance of selecting appropriately aligned group-level variables when analyzing group-level trends in ecological studies. Excluding macro-level variables could overlook crucial factors, such as trends in macroeconomic indicators that undeniably influence broader patterns. From this perspective, AJOAR, the older population, and mean temperature are suitable variables for analyzing national annual trends in ambulance dispatches, as each exhibits its own national annual trend. Conversely, the absence of individual-level data is not a major concern in an ecological study. Applying individual-level inference to group-level trends may not be appropriate, as theories relevant at the individual level do not necessarily hold at the population level. We have incorporated this reference and expanded the explanation in the Methods section to clarify the rationale behind our variable selection. (Lines 136-139, 519-520)

Regarding the validity of mean temperature, we have already cited reference 10, which demonstrated an association between mean temperature and the number of ambulance dispatches. This study, conducted in China from 2015 to 2016, found that ambulance dispatches were lowest at a mean temperature of 22.5°C and increased as the temperature deviated from that point, forming a U-shaped relationship. It also showed that replacing mean temperature with minimum or maximum temperature yielded similar results. Additionally, we have added another reference (reference 11) that further supports the association between mean temperature and the number of ambulance dispatches. (Lines 43, 154, 483-485)
One key reason for the simplicity of our model is that, among the variables previously reported to increase ambulance dispatches, only AJOAR, the older population, and mean temperature were national annual group-level variables that corresponded with national annual ambulance dispatch trends. As discussed above and stated in the Methods section, these were the most appropriate variables for inclusion (Lines 136-139). Additionally, it is generally accepted that the number of explanatory variables should be limited to one-tenth of the outcome data points to ensure the reliability of regression analysis. While this point had already been mentioned in the Methods section, we have now added a reference that explains the rationale behind this limitation (Lines 156, 542-547). Additionally, previous studies have also employed simple models. For instance, Wang et al. (reference 10) performed a regression analysis using only mean temperature as an explanatory variable in their main analysis without considering any confounders, though they conducted subgroup analyses based on gender, age group, and initial diagnosis category. An et al. (reference 11) incorporated mean temperature while adjusting for confounding factors such as seasonal trends, meteorological conditions, air pollutant levels, and day of the week, but did not account for demographic characteristics. Compared to these studies, our model incorporates a broader range of variables. We have clarified this point in the Methods section. (Line 154)

As discussed, we have added references and further discussion to clarify the validity of our methodology. We hope this addresses your concerns.

**Question:** *4. The authors have stated that they could not obtain individual patient characteristics "due to the fully anonymized data provided by government offices." I believe the precise reason is that the data was already aggregated by government offices rather than being fully anonymized, which would suggest that the data was individual but anonymized.*

**Answer:** We agree with your suggestion. The section containing the sentence has been revised as recommended. (Lines 429-430)

Dear Reviewer #1,

Thank you very much for your careful consideration of our article.

**Question:** *To further enhance the clarity and impact of your manuscript, I suggest emphasizing the significance of the TOPIX sensitivity analyses in the Discussion section. Explicitly highlighting how the consistent results validate the role of macroeconomic trends would provide additional support for your conclusions.*

**Answer:** We appreciate your suggestion. To highlight the importance of the sensitivity analysis, we created a dedicated paragraph and clarified that our hypothesis—that deflation leads to an increase in ambulance dispatches—was validated. (Lines 303-310)

Dear Reviewer #2,

Thank you very much for your careful consideration of our article.

**Question:** *An ecological study can easily contain an ecological fallacy.*

**Answer:** We agree that the limitations related to the study design should be discussed in more detail. In the Limitations section, we have added a caution regarding the ecological fallacy, emphasizing that the results should not be interpreted as evidence of individual-level causation but remain valid in the context of population-level trends. (Lines 422-431)

**Question:** *As authors stated in line 141-145 in the revised manuscript, individual factors such as health status, comorbidities, and other health-related lifestyles, are indispensable for the multivariable regression.*

*AJOAR, older population, and low-temperature cannot fully explain annual ambulance.*

*Kamikawa’s past paper in BMJ Open should take additional review if possible.*

*Moreover, Ikeda et al.’s paper handled the individual-level analysis, not aggravated data.*

*The authors must obtain individual-level health data.*

**Answer:** In an ecological study, individual-level data are not always essential. This study does not aim to investigate why “individuals” call an ambulance but rather to examine the factors driving the “national annual” trend in ambulance dispatches. When analyzing such group-level tendencies, the absence of individual-level data is not a major concern. Instead, the key is to select appropriately aligned group-level variables.

From this perspective, AJOAR, the older population, and mean temperature are suitable variables, as each exhibits its own national annual trend. Conversely, applying individual-level inference to group-level trends may not be appropriate, as theories relevant at the individual level do not always hold at the population level.

Moreover, excluding macro-level variables would overlook crucial factors—such as trends in macroeconomic indicators—that are difficult to capture through individual-level data, even in economics, yet undeniably influence broader trends.

Technically, it is also impossible to analyze national annual trends in ambulance dispatches using only individual-level data, as such an analysis would require data from Japan's entire population of approximately 120 million people over a span of 42 years.

These points are explained in detail by Zeoli et al. Therefore, we have added this article as Reference 26 and provided further explanations in the Methods section to clarify the validity of our variable selection. (Lines 136-139, 519-520)

If you are looking for additional research utilizing aggregated data without using individual-level data, please refer to the following articles.

Reference: Ishikawa M, Hemmi O, Takimoto H, Matsumoto M, Yokoyama T. Trend of estimated participation rate by regional block, gender, and age group in the 1997-2019: National Health and Nutrition Survey in Japan. PLoS One. 2024 Mar 13;19(3):e0286169.

Reference: Hamashima C, Sano H. Association between age factors and strategies for promoting participation in gastric and colorectal cancer screenings. BMC Cancer. 2018 Mar 27;18(1):345.

Reference: Matsumoto Y, Nakai A, Nishijima Y, Kishita E, Hakuno H, Sakoi M, Kusuda S, Unno N, Tamura M, Fujii T. Absence of neonatal intensive care units in secondary medical care zones is an independent risk factor of high perinatal mortality in Japan. J Obstet Gynaecol Res. 2016 Oct;42(10):1304-1309.

Dear Reviewer #3,

Thank you very much for your careful consideration of our article. We understand that there are no further questions, and therefore, no additional response is necessary.
